# *Epipactis tremolsii* Seed Diversity in Two Close but Extremely Different Populations: Just a Case of Intraspecific Variability?

**DOI:** 10.3390/plants9111625

**Published:** 2020-11-23

**Authors:** Antonio De Agostini, Pierluigi Cortis, Annalena Cogoni, Roberta Gargiulo, Giuseppe Fenu

**Affiliations:** 1 Department of Life and Environmental Sciences, University of Cagliari, Via Sant’Ignazio da Laconi 13, 09123 Cagliari, CA, Italy; deagostiniantonio@yahoo.it (A.D.A.); cogoni@unica.it (A.C.); gfenu@unica.it (G.F.); 2 Royal Botanic Gardens, Kew Richmond, Surrey TW9 3DS, UK; r.gargiulo@kew.org

**Keywords:** *Epipactis*, intraspecific variability, seed ecology, seed morphometry, heavy metals

## Abstract

Analysis of the seed morphology is a widely used approach in ecological and taxonomic studies. In this context, intraspecific variability with respect to seed morphology (size, weight, and density) was assessed in two close *Epipactis tremolsii* Pau. populations sharing the same ecological conditions, except for the soil pollution distinguishing one of them. Larger and heavier seeds were found in plants growing on the heavy metal polluted site, while no differences in seed density were detected between seeds produced by plants growing on the contaminated and the control site. Moreover, seed coats and embryos varying together in their dimensions were described in the control population, while coats varying in their size independently from embryos were described in plants growing on the polluted site. Seeds from the two studied populations significantly differed in several parameters suggesting that intraspecific seed variability occurred in the case study.

## 1. Introduction

Though studies on orchid reproduction usually focus on plant-pollinator interactions and on the role of flowers in reproductive fitness [1,2,3,4,5], reproduction in Orchidaceae is also peculiar because of the unique structure of their seeds. The development of seeds in orchids requires a very low energetic investment, as they lack endosperm (or present a very limited amount of it) and are only constituted of a small embryo contained in a membranous coat. Consequently, seeds can be produced in large amounts, reaching thousands of units in each fruit [6]. As the fruit dehisces, thousands of seeds are dispersed in the environment, mainly transported by wind, until they reach a substrate suitable for their germination. The above-mentioned, extremely simplified seed structure, together with its small size (the average length ranging from 500 to 900 µm, the smallest measuring around 100 µm) [7], could be the reason why orchid seed traits and their relationship with the environment have been overlooked in different species. To our knowledge, studies focusing on orchid seed structure and diversity generally address taxonomic questions (which comparative seed morphology could help solve) [8,9], describe the role of seeds in orchid reproduction and fungal symbiosis [10], and assess the variability of seeds in relation to growth conditions and growth habits [9,11]. Results show how seeds may vary considerably in some genera of Orchidaceae, in relation to both growth habits and taxonomy [8,9,10,11]. Nevertheless, the key role of seeds in orchid reproduction and species dispersal suggests the urgency to deepen research on their inter- and intraspecific variability, their functional aspects and their variability in relation to ecological growth conditions. In this sense, seed morphometry often represents a cheap and simple approach [12] to collect a great amount of data that can be used to address taxonomic, functional, and ecological issues [9,10,11]. Seed traits have been used in both ecological and taxonomic studies, especially in the genus *Epipactis* Zinn [13,14,15,16]. Species boundaries in *Epipactis* are difficult to define [13,17] and its taxonomy is controversial at the point that the taxonomic treatment of some taxonomic units may vary depending on different authors [17].

The present study investigates the intraspecific seed size variability in *E. tremolsii* Pau. *Epipactis* is a peculiar orchid genus because, although its ecological optimum is represented by shady forests and mature soils [18], species belonging to the genus *Epipactis* can also colonize harsh ecological contexts such as heavily anthropized habitats, thus exhibiting phenotypic plasticity [15,16,19,20,21,22,23]. Our study principally focuses on a wild population of *E. tremolsii,* which has colonized an extreme and restricted ecological context, a tailing dump, characterized by a strong heavy metal (HM) soil pollution (PPS hereafter, which stands for “plants growing on the heavy metal polluted site”) [19]. PPS present general morphological traits in common with the surrounding populations, except for the seed features, which are not easily observable in the field. Therefore, our objective was to characterize seed morphology and weight, considering their ecological and functional importance. Preliminary microscopical observations, together with the scientific literature on the topic, suggested that seeds may differ among the two studied populations depending on the peculiar growth conditions characterizing one of them (PPS) [16,24,25,26,27]. To characterize seeds produced by PPS, a second population of the same species geographically close, and sharing the same climatic and ecological conditions (except for the soil pollution), was chosen for comparison (PCS hereafter, which stands for “plants growing on the control site”). Given this framework, the present study aims to investigate the intraspecific variability in seed size and weight between two close populations of *E. tremolsii*. Specifically, the aims of this research are; (i) to determine the number of seeds fitting in a fixed volume unit as a proxy for seed size, (ii) to characterize seeds in terms of length, width and area, and (iii) to determine differences in seed weight and density in relationship to the population of origin.

## 2. Results

**Figure 1:**
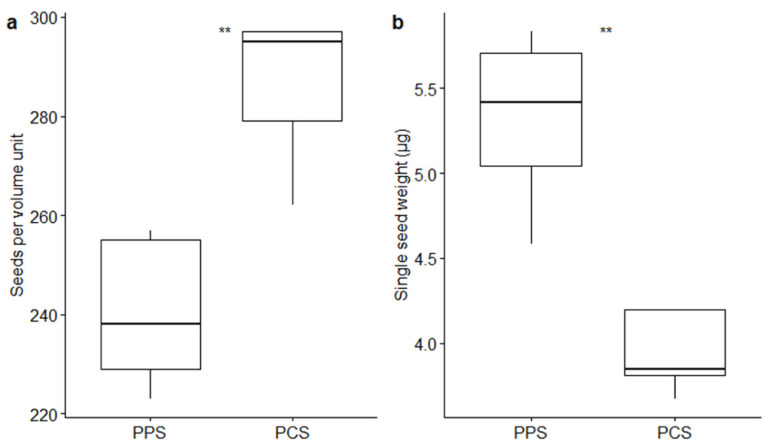
Boxplots describing differences between the number of seeds fitting in the fixed volume of 0.05 mm^3^ (**a**) and between the single-seed weight (**b**) in relation to the two different populations (PPS indicating plants growing on the polluted site; PCS indicating plants growing on the control site). Statistical significance of differences by *t*-test is reported by asterisks as follows: * = *p*-value < 0.05; ** = *p*-value < 0.01; *** = *p*-value < 0.001; **** = *p*-value < 0.0001; ns = non-significant.

Volumetric measurements pointed out a difference in seed size between the two studied populations (Figure 1a). In fact, a significantly different number of seeds (*p*-value ≤ 0.01 by *t*-test) fitted in the volume unit used in the measurements (0.05 mm^3^): 240.4 ± 6.81 (mean ± SE) seeds from PPS, whereas 286.0 ± 6.88 seeds from PCS fitted in the same volume unit. The weight (± SE) of the seeds fitting in the 0.05 mm^3^ volume unit was 1.28 ± 0.05 mg in PPS and 1.13 ± 0.03 mg in the PCS population. The mean single-seed weight was 5.32 ± 0.23 µg in PPS and 3.94 ± 0.11 µg in PCS (Figure 1b): heavier seeds were produced by PPS (*p*-value ≤ 0.01 by *t*-test). The average volume of the single seed consisted of 2.08 × 10^5^ µm^3^ and 1.75 × 10^5^ µm^3^ in PPS and PCS, respectively. The single-seed density remained relatively constant in the two populations, showing a mean value (± SE) of 2.56 × 10^−5^ ± 0.061 × 10^−5^ µg/µm^3^ in PPS and 2.25 × 10^−5^ ± 0.11 × 10^−5^ µg/µm^3^ in PCS.

**Figure 2:**
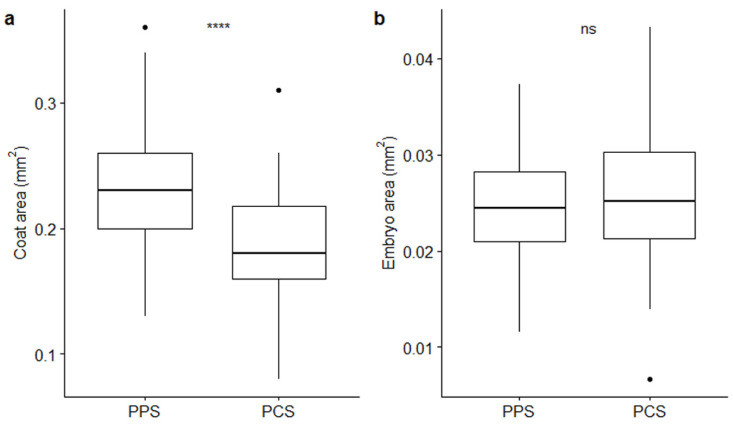
Boxplots describing differences between coat (**a**) and embryo (**b**) areas in relation to the two different populations (PPS indicating plants growing on the polluted site; PCS indicating plants growing on the control site). Statistical significance of differences (*t*-test) is reported by asterisks as follows: * = *p*-value < 0.05; ** = *p*-value < 0.01; *** = *p*-value < 0.001; **** = *p*-value < 0.0001; ns = non-significant.

The measurements of coats and embryos (Table 1; Figure 2) pointed out that coat areas vary depending on the population from which seeds come from; conversely, embryos maintained a relatively constant size. Average (± SE) coat areas were of 0.24 ± 0.005 mm^2^ and 0.19 ± 0.006 mm^2^ in PPS and PCS, respectively, with strong statistical support (*p*-value ≤ 0.0001 by *t*-test; Figure 2a), whereas average values for embryo areas were 0.02 ± 0.001 mm^2^ in PPS and 0.03 ± 0.001 mm^2^ in PCS, whereby the latter parameter did not present statistical significance, supporting the observed size differences (*p*-value > 0.05 by *t*-test; Figure 2b).

**Figure 3:**
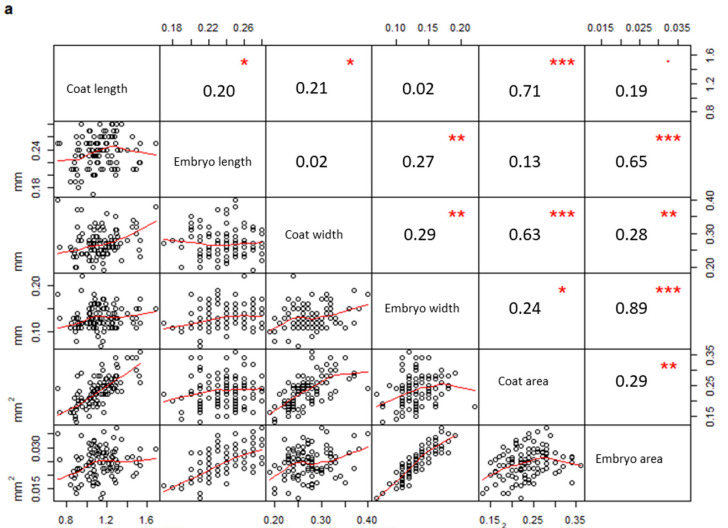

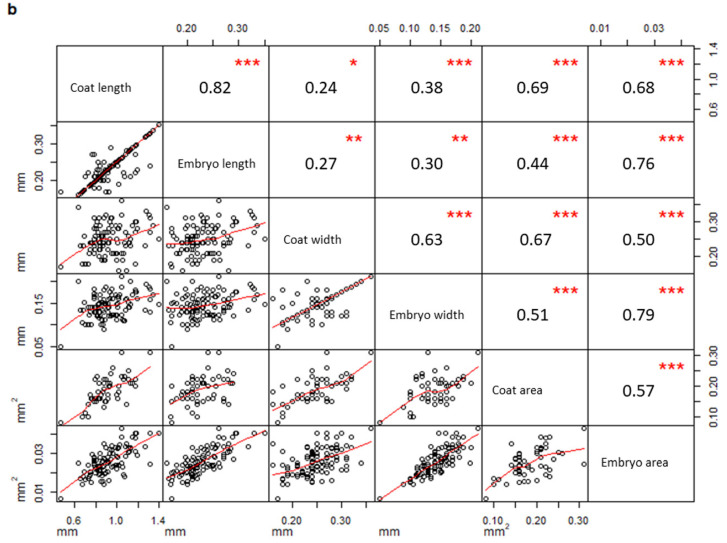
Correlation matrices describing the correlation between variables measured in PPS (**a**) and PCS (**b**), respectively (PPS indicating plants growing on the polluted site; PCS indicating plants growing on the control site). The diagonal shows the variable names, the lower half of the panel reports scatterplots between pairs of variables; the higher portion of the panel shows correlation values and their significance levels reported as asterisks. The significance of the correlations is reported by asterisks as follows: * = *p*-value < 0.05; ** = *p*-value < 0.01; *** = *p*-value < 0.001; = *p*-value < 0.1; (correlations not significant are not marked).

In addition, a correlation matrix per population was drawn considering all measured parameters. Measurements taken on seeds from PPS (Figure 3a) were significantly correlated only when they belonged to the same structure (coat or embryo), (embryo width vs embryo area, R = 0.89, *p*-value < 0.01; coat length vs coat area, R = 0.71, *p*-value < 0.01; embryo length vs embryo area, R = 0.65, *p*-value < 0.01; coat width vs coat area, R = 0.63, *p*-value < 0.01). On the contrary, several coat and embryo measurements from PCS (Figure 3b) were also correlated to each other (coat length vs embryo length, R = 0.82, *p*-value < 0.01; coat width vs embryo width, R = 0.63, *p*-value < 0.01; coat length vs embryo area, R = 0.68, *p*-value < 0.01; coat area vs embryo area, R = 0.57, *p*-value < 0.01).

## 3. Discussion

In the present study, preliminary volumetric analyses were carried out on seeds sampled from our target populations, highlighting larger seeds in PPS. A more detailed analysis focused on seed size and weight and on the relative size of embryo and coat structures pointed out that PPS produced heavier seeds with larger coats, whereas embryo size and seed density remained relatively constant in the two populations. Correlation matrices pointed out that coat and embryo sizes covaried in PCS, while coats and embryos varied independently in PPS. More precisely, embryos in PPS maintained a constant size, unlike coats, which were larger compared to PCS. Species belonging to the genus *Epipactis* can grow in a variety of natural habitats, preferring shady and humid ones, nevertheless, they can often be found settling in habitats with a strong anthropogenic disturbance, such as parks, city gardens, and areas interested by past mining activities [15,16,19,20,21,22,23]. Therefore, HM pollution of the growth substrate does not appear as a limiting factor for the distribution of this taxon in new ecological niches as a pioneer.

Seed morphometry is a widely used approach to taxonomic issues and allowed us to detect several significant differences between the two studied populations. Results obtained in the present study described intraspecific variability (regarding the analyzed seed traits), depending on the population of origin of the sampled *E. tremolsii* plants. Larger coats and embryos were found in populations inhabiting habitats with anthropogenic pressure in Rewicz et al. [16], and the authors proposed that phenotypic plasticity may have a key role in *Epipactis*, which tends to successfully colonize a wide variety of habitats. Miura et al. [10] reported the role of orchid seed coat in protecting the embryo against pathogens and non-symbiotic fungi and in harboring symbiotic fungi so that embryo infection, required for its germination, could happen in a proper way. On the other hand, the role of soil fungi in the mitigation of HM-related stress in plants has been largely investigated [28,29,30,31,32,33,34] demonstrating how soil fungi and mycorrhizal symbiosis could reduce pollutant bioavailability and protect plants against the detrimental effect of inorganic pollutants [34,35,36]. HM phytotoxicity could be extremely high in the first stages of the life-cycle (i.e., seed germination and protocorm formation) [37,38,39,40,41], given the lack of physical, physiological, and ecological mechanisms adopted by plants to prevent damage caused by soil pollutants. A potential role of the here described larger seed coats produced in PPS could be shielding embryos in their early development stages from HM pollution by harboring fungi able to reduce pollutant bioavailability to the embryo. Nevertheless, to assess whether seed coats could accomplish an adaptive ecological role protecting embryos from soil pollution, further microstructural investigations are needed. In this sense, it will be crucial to investigate the possibility of a differential pollutants’ location between seeds coats and embryos and, contextually, the role of the coat in shielding embryos from soil pollutants.

The taxonomy in *Epipactis* is still controversial, and the taxonomic treatment of entities belonging to this genus results is often difficult [17]. Nevertheless, seed size diversity alone does not allow to propose a taxonomic split nor the identification of an *E. tremolsii* ecotype corresponding to PPS. To address this issue, further studies are needed to evaluate whether observed seed intraspecific variability is a common feature of the *Epipactis* species, or it is an indication that PPS could be identified as a different taxonomic unit. In this sense, it will be crucial to extend this approach based on seed morphology studies to more *Epipactis* populations in an extensive taxonomic investigation on the genus in Sardinia. If seed variability observed in the present study should remain confined to the extreme ecological context in which PPS grew, the peculiar *E. tremolsii* population settling on the “Barraxiutta” mining tailings dump would deserve to be preserved as a genetic diversity reservoir (as reported in other research on small plant populations worldwide [42,43,44]), and as an example of adaptive strategies put in place by orchids to cope with extreme HM pollution.

## 4. Materials and Methods

### 4.1. Species Description

In Sardinia, the genus *Epipactis* is represented by six entities among species and subspecies [45]. We refer to [46], which considers the study species as *E. tremolsii*. *E. tremolsii* is a perennial (rhizomatous) herbaceous plant, producing 1 to 6 aerial stems up to 60 cm tall [18]. The inflorescence can host up to 50 opening, cross-pollinating flowers per plant. The above-described features may vary considerably in terms of size and general appearance due to the species’ tolerance to different ecological conditions [18]. Mid-shady to shady contexts on moist, deep substrates represents typical habitats of the species that frequently occur on dense woodlands edges and clearings up to 900 m a.s.l. [18].

### 4.2. Data Collection

The present study was carried out in the municipality of Domusnovas (South-West Sardinia, Italy) that hosts the two studied populations. PPS were located on a strongly HM-polluted mining tailings dump (55.98 ± 7.44 mg g^−1^ of Fe; 13.10 ± 2.71 mg g^−1^ of Zn; 5.21 ± 0.69 mg g^−1^ of Pb, data reported as mean values ± standard deviation in mg g^−1^ [19], Table S1) called “Barraxiutta”, which originated during the intense mining activity carried out in the area from 1871 to 1971 aiming to extract Pb and Zn [47]. Soil pollution characterizing the tailing dump resulted in a pollutant content in plants’ epigeal organs of 15.36 ± 4.83 mg g^−1^ of Fe, 8.52 ± 3.62 mg g^−1^ of Zn, and 1.66 ± 1.43 mg g^−1^ of Pb (data reported as mean values ± standard deviation in mg g^−1^ [19], Table S2). Conversely, PCS grew in a forested site, geographically closest to the contaminated one (c. 1.00 km as the crow flies) and was characterized by pristine conditions since no mining activity that could have generated soil pollution was ever carried out there nor in its vicinity. Moreover, the control site was chosen on a different hydrographic basin so that pollutants’ washout from the mining area could be excluded. In addition, the condition of high naturalness characterizing the control site, together with the absence of evident signals of phytotoxicity affecting the other plants growing in this site, were considered as indicative of the absence of heavy metal soil contamination. A preliminary survey was conducted to choose the control population, which was selected so that it was certainly ascribable to the same taxon inhabiting the contaminated site (Figure S1). The control and contaminated population were located a small distance apart (the control population was the *E. tremolsii* population that settled closer to the contaminated one) and shared the same ecological and climatic conditions, a shady Mediterranean *Quercus ilex* L. forest presenting mature and deep soils. Despite the fact that the tailing dump presented a scarce vegetal coverage due the soil pollution, PCS grew in a forest clearing, so that light conditions were also comparable in the two studied populations.

Seed-containing fruits were collected in the early summer (July) 2020. One mature fruit per individual, presenting early signs of dehiscence, was selected and carefully removed from the inflorescence of twenty individuals per studied population that were randomly selected. Each collected fruit was preserved in separate 50 mL-Falcon tubes and stored under controlled laboratory conditions until performing the analyses. Seed measurements were carried out separately on mixtures of seeds coming from different fruits. At first, to broadly assess differences in seed volume depending on the population of origin, volumetric measurements were carried out on the collected seeds of both populations. The number of seeds that fitted in a fixed volume of 0.05 mm^3^ (a laboratory micro spoon) was counted using a stereomicroscope. The count was repeated five times, each time using different seeds to obtain results in five replicates per population. Single-seed volumes were obtained by dividing the volume of 0.05 mm^3^ by the amount of seeds fitting in it. The single-seed weight was obtained by calculating (in 5 replicates, each one consisting of 0.05 mm^3^ × 10 volumes of seeds) the weight of 0.05 mm^3^ of seeds weighed using the Gibertini Europe 60 (Italy) analytical scale, and then dividing the obtained value by the average amount of seeds fitting in the same volume, as previously calculated. Single-seed density was consequently obtained by dividing the average single-seed weight by the average single-seed volume, as previously determined for both populations.

**Figure 4:**
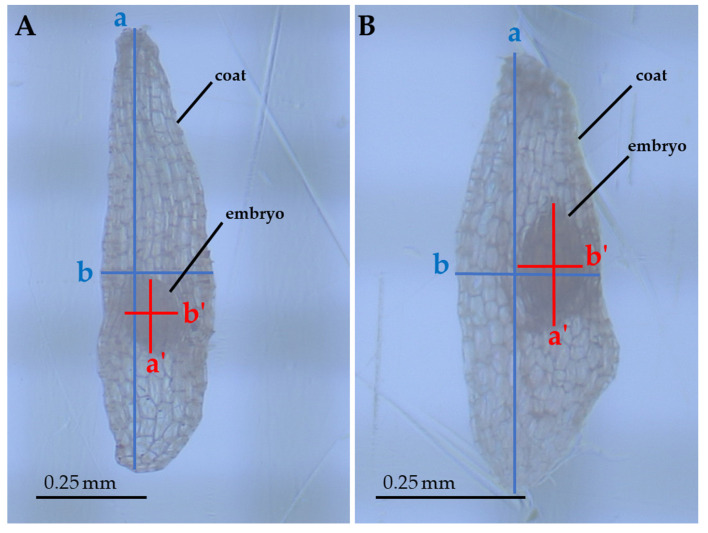
Seeds of *E. tremolsii* collected from PPS and PCS (**A** and **B,** respectively), magnified ≈ 70 times. The axes show the measurements carried out on seeds: a, coat length; b, coat width; a’, embryo length; b’, embryo width (areas were automatically calculated by the measurement software on the basis of the manually-selected perimeter of the structure).

Several linear and bidimensional seed traits were measured: Length (corresponding to the major axis), width (corresponding to the minor axis), and area (automatically calculated by the measurement software on the basis of the manually-selected perimeter of the structure) of both coats and embryos (Figure 4). These measurements allowed us to assess how the two structures (coat and embryo) varied with respect to each other. These measurements were carried using a stereomicroscope connected to a monitor by mean of the HD cam TiEsseLab TrueChrome HD IIS (Tiesselab, Italy) and using the software TiEsseLab IS CAPTURE Rel. 3.6.7 (Tiesselab, Italy). A total of 100 seeds from each population were used to carry out these measurements. All measurements were carried out during the same day, to prevent dehydration or hydration of samples from affecting measurements.

### 4.3. Data Analysis

Prior to performing any statistical analysis, we checked whether data resulting from seed measurements were normally distributed by using the Shapiro–Wilk’s test (data presenting Shapiro–Wilk’s test *p*-value > 0.05 or <0.05 were considered normally distributed or not-normally distributed, respectively), combined with a visual inspection of data distribution in case of small-sized samples (Q-Q plot). Once proved the normal or not-normal distribution of data, parametric *t*-test, and non-parametric Mann–Whitney U test were used, respectively, to assess the significance of mean differences between measurements taken on seeds coming from the two studied populations. Correlation matrices were used to better understand the relationship between the coat and embryo sizes (length, width, and areas) in relation to the two populations. Descriptive statistics were obtained using the 1.2.1335 base version of the R-Studio software [48]. The same version of the software was used to assess data distribution and test the statistical significance of the difference between paired datasets (base version), to draw plots (“ggpubr” package [49]) and to perform and plot the correlation matrix of data (“PerformanceAnalytics” package [50]), later modified concerning the font size, to improve its readability.

## Figures and Tables

**Table 1:** Measurements taken on seeds sampled from PPS and PCS (PPS indicating plants growing on the polluted site; PCS indicating plants growing on the control site). Values are reported as mean ± SE. Statistical significance of differences is reported by asterisks as follows: * = *p*-value < 0.05; ** = *p*-value < 0.01; *** = *p*-value < 0.001; **** = *p*-value < 0.0001; ns = non-significant.

Measurements	PPS	PCS	Test	Test Results
Coat length (mm)	1.13 ± 0.02	0.94 ± 0.02	Mann–Whitney U test	W = 8846; *p* = 1.06 × 10^−5^ ****
Embryo length (mm)	0.24 ± 0.002	0.23 ± 0.004	Mann–Whitney U test	W = 6281.5; *p* = 0.13, ns
Coat width (mm)	0.27 ± 0.004	0.25 ± 0.004	Mann–Whitney U test	W = 7204.5; *p* = 3.61 × 10^−4^ **
Embryo width (mm)	0.13 ± 0.002	0.14 ± 0.003	Mann–Whitney U test	W = 3904.5; *p* = 1.21 × 10^−4^ ***
Coat area (mm^2^)	0.24 ± 0.005	0.19 ± 0.006	*t*-test	t = 6.07; *p* = 5.79 × 10^−4^ ****
Embryo area (mm^2^)	0.02 ± 0.001	0.03 ± 0.001	*t*-test	t = -1.83; *p* = 0.07, ns

## Supplementary Materials

The following are available online at https://www.mdpi.com/2223-7747/9/11/1625/s1, Figure S1: Plants belonging to the two studied populations, Table S1: Heavy metal concentration levels in the tailing dump that hosts the contaminated population, Table S2: Heavy metal concentration levels in the organs of plants growing on the contaminated site.
