# Supplementary material for: Epipactis tremolsii Seed Diversity in Two Close but Extremely Different Populations: Just a Case of Intraspecific Variability?
Source: Plants (Basel). 2020 Nov 23;9(11):1625. doi: 10.3390/plants9111625 (PMC7700554; doi:10.3390/plants9111625)
Supplement: Supplementary file 1 [file plants-09-01625-s001.pdf]

Epipactis tremolsii seed diversity in two close but extremely different populations: just a case of intraspecific variability?

Antonio De Agostini [1], Pierluigi Cortis [1,*,†], Annalena Cogoni [1], Roberta Gargiulo [2], and Giuseppe Fenu [1]

1 Department of Life and Environmental Sciences, University of Cagliari, Via Sant'Ignazio da Laconi 13, 09123, Cagliari, CA, Italy;
2 Royal Botanic Gardens - Kew Richmond, Surrey TW9 3DS, UK;
* Correspondence: pierluigi.cortis@unica.it;
† Shared first co-authorship.

To follow the supplementary material to the article.

Figure S1 shows plants belonging to the two studied populations.

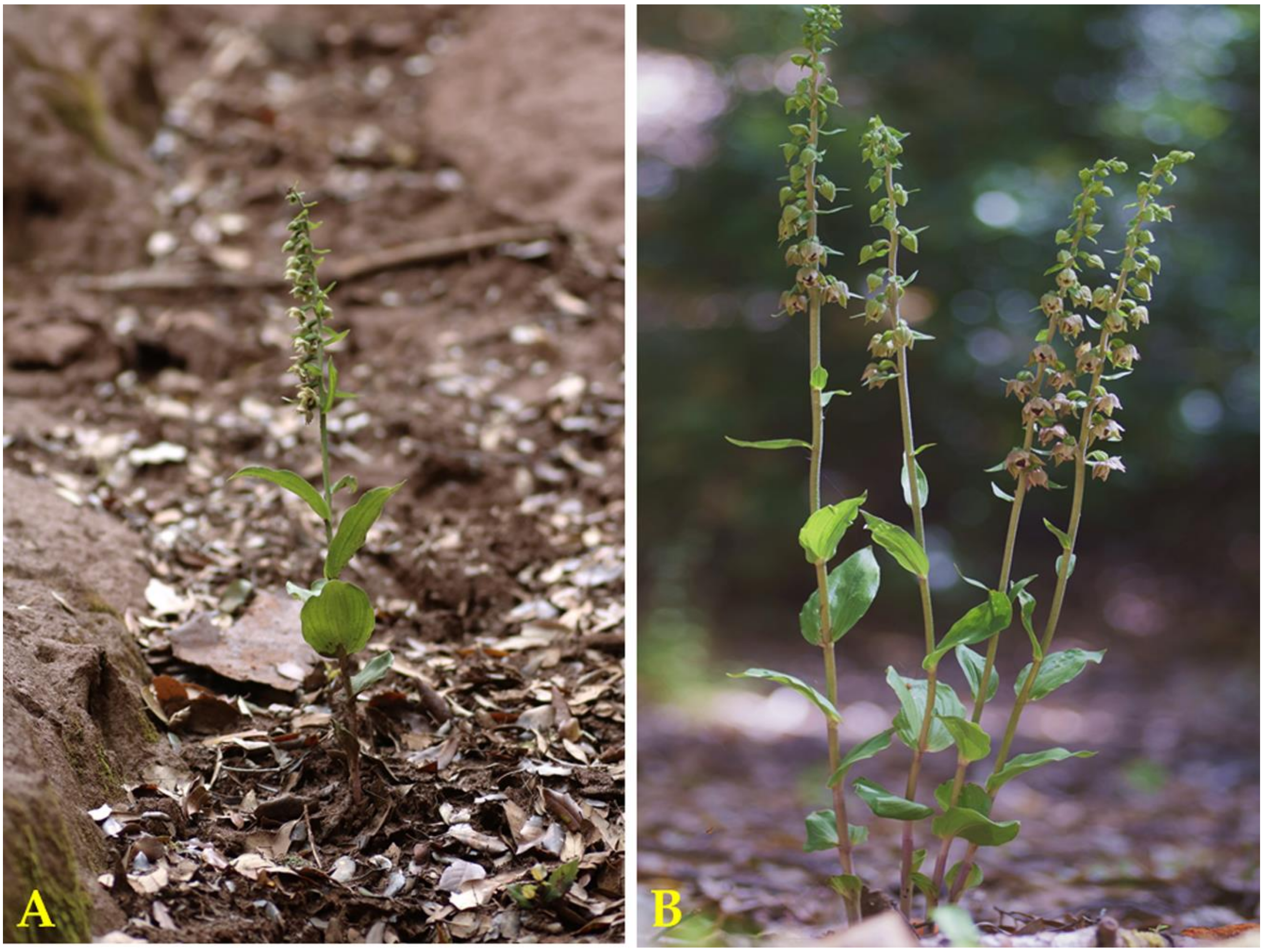

Figure S1: Plants growing in the contaminated population (A) and in the control population (B).

Table S1 reports heavy metal concentration levels in the tailing dump that hosts the contaminated population (PPS in the text); data originally reported in [1] ([19] in the main text). Table S2 reports heavy metal concentration levels in the organs of plants growing in the contaminated site (PPS in the text); data originally reported in [1] ([19] in the main text).

Table S1: heavy metal concentration levels in the tailing dump. Data are reported as mean values (mg g^{-1}) ± standard deviation.

**Element**	**Concentration**
**Fe**	55.98 ± 7.44
**Zn**	13.10 ± 2.71
**Pb**	5.21 ± 0.69
**Mn**	1.24 ± 0.06
**Cu**	0.79 ± 0.08

Table S2: heavy metal concentration levels in stem, leaves and root. Data are reported as mean values (mg g^{-1}) ± standard deviation.

**Element**	**Stem + Leaves**	**Root**
**Fe**	15.36 ± 4.83	43.08 ± 23.19
**Zn**	8.52 ± 3.62	23.28 ± 14.15
**Pb**	1.66 ± 1.43	4.10 ± 2.50
**Cu**	0.26 ± 0.84	0.09 ± 0.06

References

1. De Agostini, A.; Caltagirone, C.; Caredda, A.; Cicatelli, A.; Cogoni, A.; Farci, D.; Guarino, F.; Garau, A.; Labra, M.; Lussu, M.; Piano, D.; Sanna, C.; Tommasi, N.; Vacca, A.; Cortis, P. Heavy metal tolerance of orchid populations growing on abandoned mine tailings: A case study in Sardinia Island (Italy). *Ecotoxicol. Environ. Safety* **2020**, 189, 110018. https://doi.org/10.1016/j.ecoenv.2019.110018
